# CHL1-Deficient and Wild-Type Male Mice do Not Differ in Locomotor Recovery from Spinal Cord Injury

**DOI:** 10.26502/fjsrs0047

**Published:** 2022-09-20

**Authors:** Thomas Theis, Carlos Ayala, Monica Tschang, Michele Philip, Vini Nagaraj, Anil Shrirao, Gregory Voronin, Wise Young, Melitta Schachner

**Affiliations:** 1 Keck Center for Collaborative Neuroscience and Department of Cell Biology and Neuroscience, Rutgers University, Piscataway, NJ 08554, USA; 2 Department of Biomedical Engineering, Rutgers University, 599 Taylor Road, Piscataway, NJ 08854, USA; 3 In Vivo Research Services Core, Rutgers University, 604 Allison Road, Piscataway, NJ 08854, USA

**Keywords:** Close homolog of L1, Spinal cord injury, Sex differences, Inflammation, Locomotor recovery

## Abstract

CHL1 is a close homolog of L1, a cell adhesion molecule that plays major roles in neural and tumor cell functions. We had found that young adult female mice deficient in CHL1 recovered better than their wild-type female littermates after thoracic Spinal Cord Injury (SCI). This observation was surprising, because CHL1 increases neurite outgrowth in vitro. Injury of adult mouse central and peripheral nervous systems upregulate CHL1 expression in neurons and astrocytes, consistent with CHL1’s pro-active, homophilic interaction between CHL1 surface molecules in wild-type mice. After SCI, CHL1 expression was observed to increase in the glial scar, areas of axonal regrowth and remodeling of neural circuits. These observations were made only in females, and we therefore sought to analyze SCI in CHL1-deficient male mice. We now show that CHL1-deficient males did not recover better or worse than their male wild-type littermates. Primary and secondary lesion volumes were similar in the two genotypes, as seen in female mice which were studied in parallel with male mice. Assessment of peripheral leukocytes showed a significant increase in numbers of blood neutrophils at 24 h after SCI in males, but not in females. Lymphocyte numbers in mutant males increased slightly, but numbers of lymphocytes or monocytes did not differ significantly between males or females. These results indicate that CHL1-deficient males and females differ in the number of neutrophils but not lymphocytes or monocytes, suggesting that the difference between males and females is unlikely due to differences in leukocytes.

## Introduction

Cell Adhesion Molecules (CAMs) play important roles in nervous system during development, synaptic activity and plasticity, and regeneration after trauma in adulthood [1–3]. The immunoglobulin superfamily molecule L1CAM/CD171, here shortened to L1, was the first CAM that improved recovery after spinal cord injury (SCI) in rats [4] and mice [5], when administered to injured spinal cords. Other members of the immunoglobulin superfamily of CAMs contribute to functional recovery after SCI in rats, zebrafish, and other species [6]. Like L1, mutations of close homolog of L1 (CHL1, designated as ‘CALL’ in humans) are linked to neurological disorders that include psychiatric and other manifestations, including mental retardation [7], schizophrenia [8], and autism [9]. Similar to L1, CHL1 is a transmembrane glycoprotein with immunoglobulin-like and fibronectin type III homologous domains [10]. In vitro studies indicated that CHL1 promotes neurite outgrowth [11] and neuronal survival [12], as well as enhances neuronal cell migration [13]. Unlike L1, however, CHL1-CHL1 homophilic (i.e. self-binding) interactions inhibit neurite outgrowth as seen in neuron/astrocyte co-culture experiments [14]. In vivo experiments with CHL1-deficient female mice confirmed that that the presence of CHL1 suppresses regeneration after spinal cord injury [14]. However, the influence of CHL1 on the consequences of SCI in males was not studied at the time, because females were traditionally considered to be more conducive to be taken care of after surgery and for measurements of locomotor recovery, In the present study, we investigated the response of male CHL1-deficient (CHL1−/−) mice to SCI. We report that, in contrast to female CHL1−/− mice, these mice did not show an improved recovery of locomotion after SCI when compared to their wild-type (CHL1+/+) male littermates. Unexpectedly, locomotor recovery did not statistically differ between male mutant and wild-type genotypes. Also, primary, and secondary lesion volumes were similar. Similarly, comparable numbers of Iba1-immunopositive cells occupied similar volume sizes at the lesion site. In search for immune system-related differences, we found that male, but not female mutant mice had increased numbers of inflammatory blood neutrophils, suggesting that they are important players in regeneration after mouse SCI

## Materials And Methods

### Animals

The Institutional Animal Care and Use Committee of Rutgers University approved all experiments (protocol # 09–051). All experiments used 9- to 12-week-old C57BL/6J mice (CHL1+/+, n = 8 males and 4 females) and CHL1-deficient (CHL1−/−, n = 8 males and 4 females) littermates from heterozygous breeding [15]. Mice were maintained and bred with ad libitum access to food and water on a 12-hour light and 12-hour dark cycle. The average weights ± SD were for male CHL1+/+ 28 g ± 3 g, for male CHL1−/− 29 g ± 4 g, for female CHL1+/+ 25 g ± 1 g, and for female CHL1−/− 26 g ± 3 g.

### Polymerase chain reaction

A tail snip from each mouse was tested with polymerase chain reaction (PCR) to confirm CHL1 genotype. DNA was extracted with Bioline MyTaq^™^ Extract-PCR Kits (cat# BIO-2112). The CHL1 gene was amplified with primers CHL1-A(forward:AATTGATCGAGGCAGCACTACTTTCTG),HL1-5’up(reverse:TTCCCAGAAAGGAGGCUSAAACGTG) and NeoBosl (reverse: CTAAAGCGCATGCTCCAGACTGCC) (DNA Technologies, Iowa, USA). A thermal cycler (Mastercyler Ep-gradient, Eppendorf, Hamburg, Germany) was used to amplify genes in the following sequence: 1.5 minutes at 95°C; 2. 40 cycles of 15 seconds at 95°C, 15 seconds at 63.5°C, and 20 seconds at 72°C. Samples were then cooled to 4°C.

### Antibodies and reagents

Chemicals were obtained from Sigma-Aldrich (St. Louis, MO, USA) if not indicated otherwise. The following antibodies were used for immunohistochemistry to quantify the lesion volume, the lack of myelin surrounding the lesion, and volume of accumulated microglia and monocytes in the core of the lesion, respectively: Anti-glial fibrillary acidic protein (GFAP) antibody, clone GA5, was from Sigma-Aldrich (cat# MAB360), anti-myelin basic protein (MBP) was from Millipore Sigma (cat# MAB386, Burlington, MA, USA), and anti-ionized calcium binding adaptor molecule 1 (Iba1) antibody clone [EPR16588] was from Abcam (cat# ab178846; Cambridge, UK), All secondary antibodies were from Jackson ImmunoResearch (Pennsylvania, USA).

### Spinal cord injury

Mouse spinal cords were severely contused at T11 as described [16]. In brief, mice were anesthetized with isoflurane, initially at 5%. Anesthesia was then maintained at 2% during surgery. The upper-back skin was shaved, disinfected three times, and incised to expose the vertebral column. A laminectomy at T9-10 vertebral level exposed the spinal cord and the adjacent spinous processes were secured with clamps. The spinal cord was contused at T11 with a 5-g weight-drop from a 6.25 mm height using the Multicenter Animal Spinal Cord Injury Study Impactor device [16]. After contusion, paravertebral muscles adjacent to the laminectomy site were sutured and the skin was closed with 9 mm wound clips (cat# RS-9260; Roboz Surgical Instrument Co, Maryland, USA). Shortly after surgery and on the first day thereafter, the mice received one 5 ml or 10 ml of subcutaneous saline injection for females and males respectively, followed by 25 mg / kg Cefazolin (cat# 054846; Covetrus, Portland, ME, USA). Injured mice were single-housed in cages with fresh bedding and maintained on a heating pad overnight after surgery. Mice were monitored for autophagy and infections, and urine was expressed twice daily 48 hours after SCI and then once daily until mice had empty bladders in the morning, indicating that they were able to urinate.

### Locomotor recovery

The recovery of locomotor function after SCI was analyzed using the Basso Mouse Scale (BMS) [17] and single frame motion analysis [14]. For BMS scores, spinal cord injured mice were placed in an open-field for 5 minutes and two researchers graded to consensus the coordination of stepping and hind-limb joint movements in both legs at 2 days and 1, 2, 3, and 4 weeks after SCI. A score of 0 represents no hind-limb movement, scores increase with better hind-limb joint coordination, and a score of 9 represents normal walking.

The extension-flexion ratio, foot-stepping angle, and rump-height index were estimated by single frame motion analysis [14]. Spinal cord injured mice were recorded with a Hero3 camera (cat# CHDNH-B10; GoPro, California, USA) while running on a treadmill (speed: uninjured, 20 cm/sec; injured, 2.5–10 cm/sec) and single frame motion analysis was used to determine the foot-stepping angle and rump-height index as described [14]. The foot-stepping angle is measured with respect to the posterior aspect at the beginning of the stance phase. In uninjured mice, the angle in this phase is approximately 30° [14]. The foot-stepping angle increases after SCI and decreases with recovery. The average of values was taken from three to five measurements per mouse at each time point. The rump-height index was assessed from the same recordings that were used for measurements of the foot-stepping angle. The vertical distance from the animal’s tail base to the ground of the treadmill was normalized to the width of the slit in the clear back panel of the treadmill construction. The rump-height index reflects the ability to support body weight and it decreases after SCI.

The extension-flexion ratio test measures voluntary movements without body weight support. A researcher holds a spinal cord injured mouse by its tail and allows it to grasp a pencil with its forepaws (“pencil test”). The cycling extension-flexion movement of the hind-limbs was recorded from the left side view during the time that the mouse was trying to catch the pencil with its hind-paws. The distance from the most distal midpoint of the paw to the tail base was measured during maximal extension and maximal flexion, and the ratio was calculated. This ratio decreases after SCI and increases with recovery.

Video recordings were viewed in single frame with the VLC media player SM Play (GNU GPL 2.0 license, http://www.gnu.org/licenses/gpl-2.0.html) and ImageJ (Version 1.50b; National Institutes of Health) software.

### Immunohistochemistry

At 4 weeks after SCI, mice were deeply anesthetized with 5% isoflurane and transcardially perfused with 10 U/mL heparin saline for 2 minutes followed by incubation in 4% formaldehyde diluted in saline for 10 minutes at room temperature. The spinal cords were dissected and sagittally cryosectioned as described [16]. In brief, 1 cm of spinal cords that contained the injury site in the center was dissected, post-fixed in 4% formaldehyde in saline and cryoprotected in PBS with increasing sucrose concentrations (10, 15 and 20%). Spinal cords were then frozen in Tissue-Tek^®^ O.C.T. Compound (Sakura, cat #45834; California, USA) medium and 20-μm-thick serial sagittal section were prepared and mounted on Fisherbrand Superfrost Plus Microscope Slides (cat# 12-550-15; Fisher Scientific, Massachusetts, USA). Before immunohistochemistry, antigen retrieval was performed by incubating sections for 30 min at 80°C in 0.1 M sodium citrate (pH 6.0). Sections were then blocked with 5% BSA and 0.3% Triton-X 100 in PBS for 1 hour at room temperature. Triple staining was performed by incubating the spinal cord sections overnight at 4°C with antibodies diluted in blocking solution (Iba1, 1:1000; GFAP, 1:500, MBP 1:50). Sections were then washed three times at room temperature with PBS containing 0.3% Triton-X 100 and incubated with the corresponding Alexa Fluor 488- or Alexa Fluor 647-conjugated donkey secondary antibodies (1:1000 in blocking solution) for 1 hour at room temperature. Sections were mounted with ProLong^™^ Gold Antifade Mountant with DAPI (cat# P36931; Life Technologies, California, USA) and images were captured with an Axiovert200 Fluorescence Live Cell Imaging Workstation (Carl Zeiss). Immunofluorescence intensity of sections at and 2.5 mm rostral to the injury site were measured using ImageJ. Ratios between immunofluorescence intensity rostral to and at the injury site were calculated and averaged for each condition.

### Quantification of blood leukocytes

Mice were anesthetized with 5% isoflurane and blood samples were taken from the retro-orbital vein using heparin-coated Fisherbrand^™^ Micro Blood Collecting Tubes (cat# 02-668-10; Fisher Scientific) 24 hours after SCI. Hematological analysis was performed using the Heska ElementHT5 (Heska, Colorado, USA). The Heska ElementHT5 counts red blood cells by the electrical impedance method, and leukocytes and their subclasses —such as neutrophils, lymphocytes, and monocytes— were measured by a colorimetric method that utilize the different optical properties of the different leukocytes without any specific staining.

### Statistics

All animal treatments, data acquisition, and analyses were performed in a blinded manner. Before using the appropriate statistical comparison, normal distribution of the data was determined by Shapiro-Wilk test. Locomotor recovery after SCI was statistically analyzed by repeated measures ANOVA. For all other experiments, groups were compared by one-way ANOVA with Fisher’s protected least significant difference (PLSD) post-hoc test using StatView Version 5.0.1 (SAS Institute Inc, New York, NY, USA), and Microsoft Excel (Redmond, Washington, USA) for all calculations.

## Results

**Figure 1:**
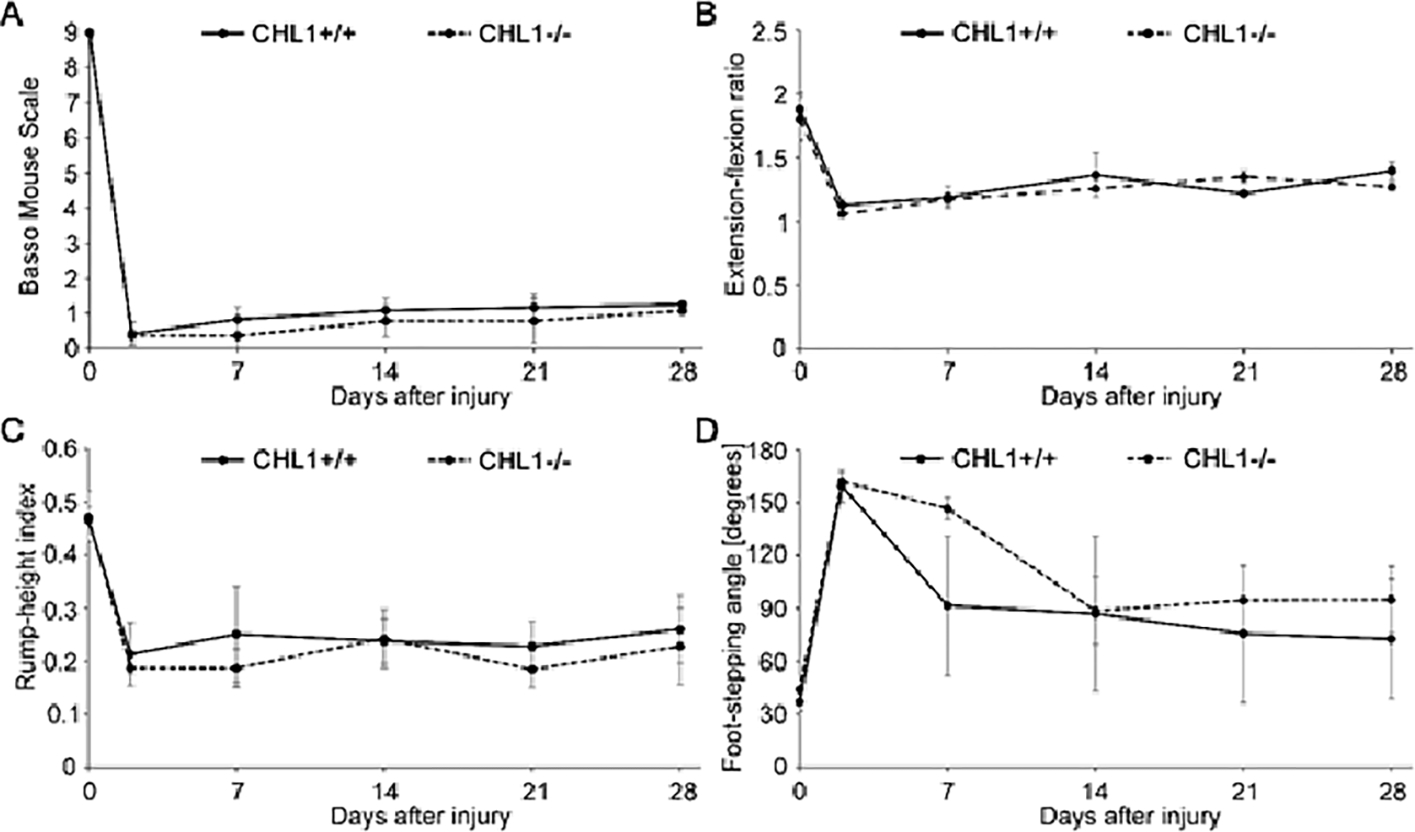
No difference in locomotor behavior of male CHL1-deficient (CHL1−/−) and wild-type CHL1 (CHL1+/+) littermate mice measured up to 28 days after SCI. Hind-limb recovery was assessed by (A) Basso Mouse Scale, (B) extension-flexion ratio, (C) rump-height index and (D) foot-stepping angle. CHL1+/+, 6 mice per group; CHL1−/−, 7 mice per group. Two-way repeated-measured ANOVA. (A: F(5, 55) = 0.289, p = 0.917; B: F(5, 30) = 0.586, p = 0.711; C: F(5, 20) = 0.214, p = 0.952; D: F(5, 20) = 0.756, p = 0.592). Error bars indicate ± SEM.

Given the interest in recovery from SCI, we started our experiments with young adult male CHL1−/− and CHL1+/+ literate controls. The locomotor behavior was measured weekly over period of 28 days after SCI (Figure 1) and showed no difference between genotypes in BMS (Figure 1A), extension-flexion ratio, also called pencil grabbing test (Figure 1B), rump-height index (Figure 1C) or foot-stepping angle (Figure 1D). These results underscore the difference in locomotor recovery observed in CHL1−/− female mice.

**Figure 2:**
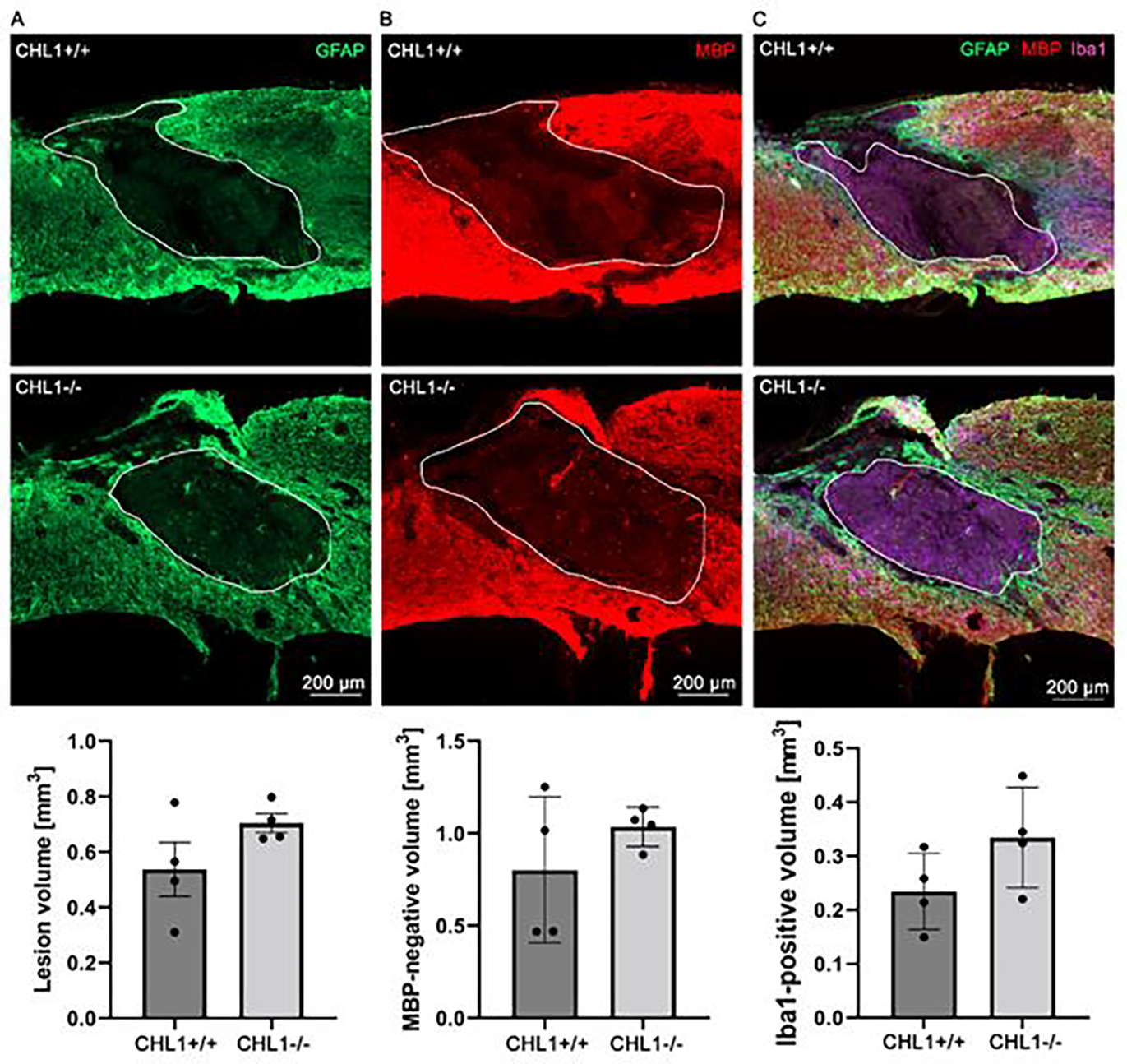
No significant differences between genotypes in lesion volume as assessed by absence of immunostainings for GFAP, MBP and Iba1 at 28 days after SCI. Representative images show triple immunostainings of (A) GFAP+ (green) (A), (B) MBP+ (red) (B), and (C) Iba1+ (purple) in sagittal sections. (A), (B) white lines delineate lesion areas, and (C) white lines circumscribe the Iba1+ area. Bar diagrams indicate average absence of immunostainings for GFAP (lesion volume), for MBP (secondary lesion volume) and volume of Iba1+ cells at the lesion sites. Student’s t-test. Error bars indicate ± SEM (4 mice per genotype).

To investigate primary and secondary lesion volumes, sagittal sections were immunostained for GFAP for astrocytes (Figure 2A), MBP for oligodendrocytes (Figure 2B), and Iba1 (Figure 2C) for monocytes that comprise macrophages and microglia. The bar diagrams below the immunofluorescence images show quantitative evaluations obtained from four animals. No significant differences could be seen.

**Figure 3:**
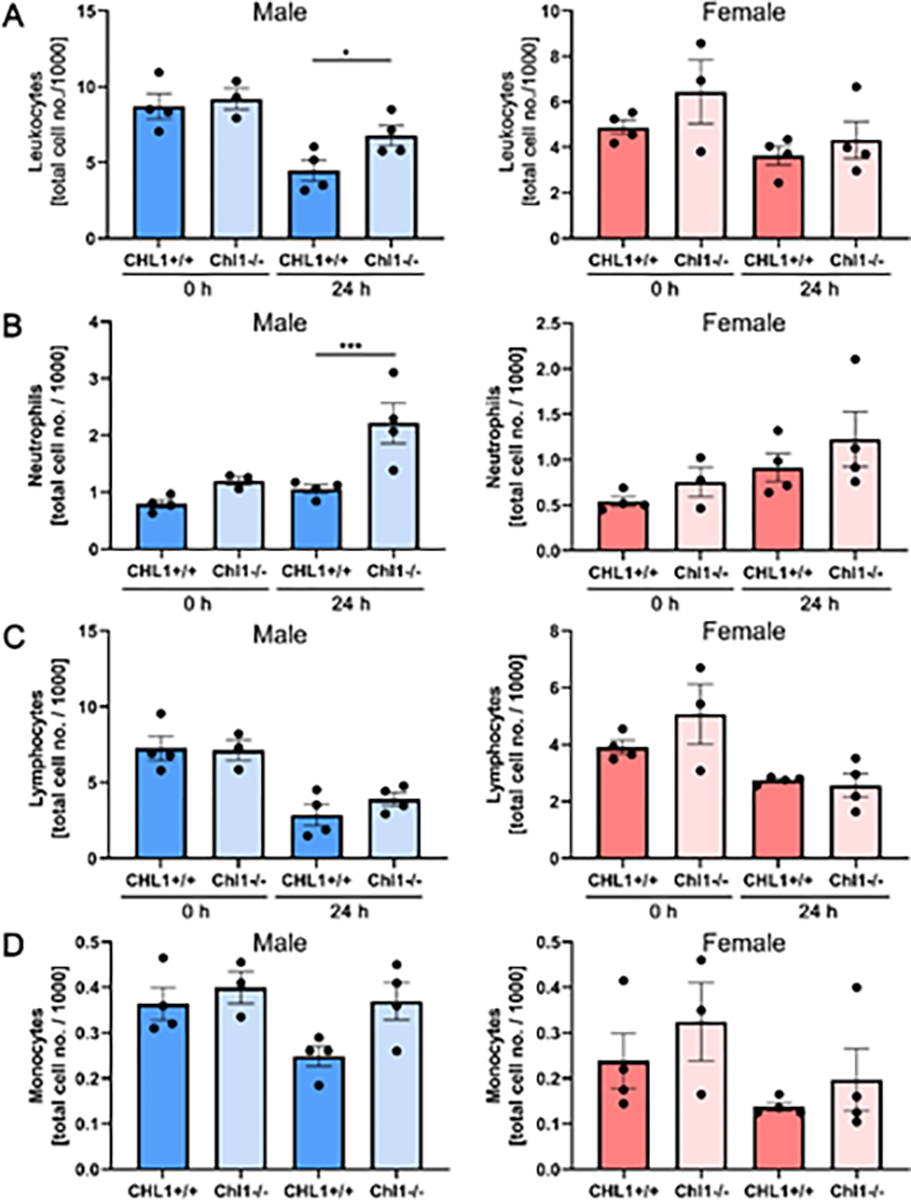
Increased numbers of blood neutrophils in male CHL1−/− mice versus wild-type littermate mice at 24 hours after SCI. Blood samples were taken from CHL1+/+ and CHL1−/− male (blue bars) and female (red bars) mice before (0 hour) and 24 hours after SCI. Total numbers of (A) leukocytes, (B) neutrophils, (C) lymphocytes and (D) monocytes were determined by the Heska ElementHT5 (4 genotypes per group). (A: F=7.844, p<0.0001; Fisher’s PLSD: *p<0.05; B: F=6.091, p=0.0002, Fisher’s PLSD: ***p<0.001). Error bars indicate ± SEM.

We next evaluated several immune system cell types in the blood of male and female mice of both genotypes (Figure 3). No differences were detected in mutant and wild-type females (Figure 3A–D, red columns). In males (Figure 3A–D, blue columns) a statistically small difference could be seen for lymphocytes (Figure 3A). A significant difference was seen for neutrophils (Figure 3B). Lymphocytes (Figure 3C) and monocytes (Figure 3D) did not show any differences and are therefore similar to the results from the females.

## Discussion

In this study, we found that CHL1−/− male mice did not show an improved functional recovery after spinal cord injury and they had no differences in the lesion volume compared to their CHL1+/+ littermates. This finding was surprising since we had published that CHL1−/− female mice had an improved functional recovery after SCI compared to their CHL1+/+ female littermates [18]. Based on previous reports mentioning an altered inflammation of CHL1−/− mice in mouse models of inflammatory bowel disease, we became interested in immune system-related functions after SCI. Also, dextran sulfate-induced colitis was reported to lead to exacerbated inflammation and damage to colonic tissues of CHL1−/− mice with an increased neutrophil and macrophage infiltration [18]. In contrast, another group reported an attenuated inflammatory response of CHL1−/− mice in a dextran sulfate-induced inflammatory bowel disease mouse model [19]. However, the two studies did not indicate the sex of mice used.

Since inflammation plays an essential role in the development of secondary damage after SCI [20], we studied the potential differences in inflammation after SCI in CHL1−/− mice of both sexes by hematological analysis. Our results show a more aggressive inflammatory response after SCI in male CHL1−/− mice compared to their wild-type female littermates as indicated by an increased number of leukocytes. More specifically, we observed an increased number of neutrophils in the blood of CHL1−/− male mice 24 hours after SCI compared to their wild-type male and female littermates.

Neutrophils, also known as polymorphonuclear leukocytes, are the first responders after SCI and are the first immune cells that infiltrate into the injured spinal cord [21,22]. Neutrophils are the largest group of leukocytes in the human blood. They are involved in host defense against invading pathogens by oxidative and non-oxidative mechanisms. Neutrophils express NADPH oxidase complexes to produce reactive oxygen species and fight pathogens. In addition, they can generate neutrophil extracellular traps which are web-like structures composed of DNA, histones, and granules. These highly sticky nets entrap and eliminate pathogens [23]. However, after SCI, these oxidative and non-oxidative mechanisms induce secondary damage and attenuate functional recovery of the spinal cord [24]. In addition, a selective inhibitor of neutrophil elastase attenuates glial scar formation and promotes functional recovery after SCI [25].

Neutrophils have a short life span of fewer than 24 hours in the bloodstream and their high turnover rate is regulated by apoptosis and clearance by macrophages [26]. An impaired regulation of neutrophil cell death is suggested to contribute to a variety of inflammatory pathologies and thus, neutrophil apoptosis is essential to regulate the timely resolution of inflammation [27]. We observed an increased number of neutrophils after SCI in male CHL1−/− mouse blood which indicates that the rapid neutrophil turnover is disturbed and leads in CHL1−/− male mice to a prolonged inflammatory response after SCI. This rapid neutrophil turnover is controlled by precursor cell proliferation and differentiation in the bone marrow, the subsequent release of mature neutrophils into the blood, a potential neutrophil margination in organs such as the lung and spleen, and the final neutrophil apoptosis [28]. CHL1-mediated interactions between neutrophils and the surrounding tissue might either regulate the differentiation and maturation of neutrophils in the bone marrow or enhances the margination in organs by neutrophil adhesion or inhibit neutrophil apoptosis. Although CHL1 is widely expressed in neurons, astrocytes, and oligodendrocyte progenitor cells in the central nervous system, CHL1 in the immune system is predominantly expressed in B-cells and not in neutrophils [29]. Thus, it is likely that heterophilic interactions between CHL1 expressed in the surrounding tissue environment and proteins localized on the cell surface of neutrophils lead to increased numbers of circulating neutrophils in CHL1−/− male mice after SCI by either regulating adhesion, differentiation, or apoptosis of neutrophils.

This observation would not explain the observed sex-specific effect since we observed an increased number of neutrophils in the blood after SCI in male but not female CHL1−/− mice. Interestingly, there are reports of sex-differences in neutrophil biology [30]. Single-cell RNA-sequencing analysis of human blood from healthy adult females and males revealed that neutrophils in males are more immature and less activated than their female counterparts. The transcriptome analysis of neutrophils isolated from females indicated an enhanced responsiveness to Toll-like receptor agonists, whereas neutrophils from males had a strongly increased mitochondrial metabolism interpreted to suggest that these sex-specific differences in neutrophil phenotypes are hormonally driven and not X-chromosome dependent. These sex-specific differences in the maturation state of neutrophils might lead to differences in the expression levels of cell surface proteins in neutrophils. Consequently, CHL1 in the tissue surrounding neutrophils would have different interaction partners in males and females, and thus, heterophilic CHL1 interactions with neutrophils would have different functional consequences in males compared to females. In consequence, the absence of CHL1 in surrounding tissue might affect the number of circulating neutrophils in males but not in females. To interpret our findings that in contrast to female CHL1−/− mice, CHL1−/− male mice do not differ between genotypes in functional recovery, we suggest that CHL1−/− male mice, like their female littermates, would show enhanced recovery in locomotion after SCI because CHL1 does not block the regrowth of axons. However, this function does not become obvious since acute neutrophil-based inflammation counteracts this CHL1-based function of successful axonal regrowth. It is interesting in this context that the total number of circulating neutrophils increases 24 hours after SCI, thereby indicating a prolonged inflammatory persistence. This observation is notable, since it has been it has been reported that neutrophils mature differently in males and females. We would thus hypothesize that male and female neutrophils express different cell surface molecules which may contribute to different functional consequences in heterophilic interactions between neutrophils and the spinal cord host tissue. These sex-specific differences in the neutrophil maturation state are likely to be hormonally driven, thus leading to the question how hormone therapy may enter clinical trials.

### Limitations

Although this study had a relatively small group size of mice for the hematological analysis, a significant increase in neutrophil numbers was observed in the group of male CHL1−/− mice. There was no tool available to us that blocks specifically the neutrophil response after SCI, so we could not test our hypothesis that the observed neutrophilia affects the functional recovery of male CHL1−/− mice after SCI. Although cyclophosphamide is in some studies used to induce neutropenia [31], it affects also other immune cells and is not specific for neutrophils. It was therefore not used in the present study.

## Conclusion

In contrast to our study on CHL1-deficient female mice [14], CHL1-deficient males did not recover better or worse than their male wild-type littermates. Primary and secondary lesion volumes were similar in the two genotypes under all conditions studied. In the peripheral blood at 24 h after SCI, neutrophil numbers were higher in males than in females. These results indicate that CHL1-deficient males and females differ in the number of neutrophils, slightly in numbers of leukocytes, but not lymphocytes or monocytes. These sex-specific differences in immune response after SCI might affect the functional recovery after SCI.

## Data Availability

The data that support the findings of this study are available from the corresponding author (M.S.) and the first author (T.T.) upon reasonable request.

## Abbreviations

BMS: Basso Mouse Scale
CAMs: Cell Adhesion Molecules
CHL 1: Close Homolog of L1
CHL1−/−: CHL1-deficient
PLSD: Fisher’s Protected Least Significant Difference
GFAP: Glial Fibrillary Acidic Protein
IBA1: Ionized calcium Binding Adaptor molecule 1
MBP: Myelin Basic Protein
SCI: Spinal Cord Injury
Wild-Type: (CHL1+/+)
